# Evidence-based health information from the users’ perspective – a qualitative analysis

**DOI:** 10.1186/1472-6963-13-405

**Published:** 2013-10-10

**Authors:** Irene Hirschberg, Gabriele Seidel, Daniel Strech, Hilda Bastian, Marie-Luise Dierks

**Affiliations:** 1 CELLS – Centre for Ethics and Law in the Life Sciences/Hannover Medical School, Institute for History, Ethics and Philosophy of Medicine, Carl-Neuberg-Str. 1, 30625 Hannover, Germany; 2 Institute for Epidemiology, Social Medicine and Health Systems Research, Hannover Medical School, Carl-Neuberg-Str. 1, 30625 Hannover, Germany; 3 U.S. National Center for Biotechnology Information, National Library of Medicine, National Institutes of Health, 8600 Rockville Pike, Bethesda, MD 20894, USA

## Abstract

**Background:**

Evidence-based information is a precondition for informed decision-making and participation in health. There are several recommendations and definitions available on the generation and assessment of so called evidence-based health information for patients and consumers (EBHI). They stress the importance of objectively informing people about benefits and harms and any uncertainties in health-related procedures. There are also studies on the comprehensibility, relevance and user-friendliness of these informational materials. But to date there has been little research on the perceptions and cognitive reactions of users or lay people towards EBHI. The aim of our study is to define the spectrum of consumers’ reaction patterns to written EBHI in order to gain a deeper understanding of their comprehension and assumptions, as well as their informational needs and expectations.

**Methods:**

This study is based on an external user evaluation of EBHI produced by the German Institute for Quality and Efficiency in Health Care (IQWiG), commissioned by the IQWiG. The EBHI were examined within guided group discussions, carried out with lay people. The test readers’ first impressions and their appraisal of the informational content, presentation, structure, comprehensibility and effect were gathered. Then a qualitative text analysis of 25 discussion transcripts involving 94 test readers was performed.

**Results:**

Based on the qualitative text analysis a framework for reaction patterns was developed, comprising eight main categories: (i) interest, (ii) satisfaction, (iii) reassurance and trust, (iv) activation, (v) disinterest, (vi) dissatisfaction and disappointment, (vii) anxiety and worry, (viii) doubt.

**Conclusions:**

Many lay people are unfamiliar with core characteristics of this special information type. Two particularly critical issues are the description of insufficient evidence and the attendant absence of clear-cut recommendations. Further research is needed to examine strategies to explain the specific character of EBHI so as to minimize unintended or adverse reaction patterns. The presented framework describes the spectrum of users’ reaction patterns to EBHI. It may support existing best practice models for editing EBHI.

## Background

Sufficient, understandable and reliable information is a precondition for citizens’ informed participation and self-determined action in health and illness. Moreover, people have the right to comprehensive information and education [1-5]. Therefore, people need unbiased and reliable information based on the current state of medical knowledge [6], so-called evidence-based health information (EBHI) [7,8].

Various national and international recommendations for generating and assessing EBHI are currently available [6,9-13]. All of them stress that people must be objectively informed about benefits and harms and any uncertainties that might exist in health-related procedures.

It is also stipulated that lay people should be integrated in the process of generating and evaluating the material, in order to increase the comprehensibility, relevance and user-friendliness of materials [9,10,14]. To date there has been little research on the spectrum of effects and reaction patterns to written EBHI [15]. Our study aims to define the spectrum of consumers’ reaction patterns to written EBHI to get a deeper understanding of their comprehension and assumptions, but also of their informational needs and expectations, and thereby, to contribute to the ongoing development of this information type.

The study is based on the results of an external user evaluation of EBHI produced by the Institute for Quality and Efficiency in Health Care (Institut für Qualität und Wirtschaftlichkeit im Gesundheitswesen, IQWiG). IQWiG is an independent scientific institute in Germany, established by legislation and commissioned by the German healthcare system or Ministry of Health. Its methods adhere to principles for evidence-based medicine (EBM). One of IQWiG’s legislative mandates is to provide EBHI for patients and the general public via the internet [9,16-18]. Besides internal evaluation and routine monitoring of the website, it also commissions external evaluations and user testing [19-21]. The user testing described here was conducted by the so called “Patient University” at Hannover Medical School (MHH) [19,22,23], by order of the IQWiG.

## Methods

### The user testing

From June 2008 to March 2009, altogether 107 of IQWiG’s health information products consisting of “fact sheets”, “research summaries“and “supplementary elements” (Table 1) were evaluated by 124 consumers in moderated focus group discussions sponsored by the IQWiG. The study was approved by the local ethics committee of Hannover Medical School (No. 1600-2012). Within our study we adhere to the RATS guidelines on qualitative research [24]. Our study was in compliance with the Helsinki Declaration.

**Table 1:** IQWiG information products tested between June 2008 and March 2009

**Product or type of text**	**Description**	**Number**
**Fact sheets**	About three to six pages of easily understandable information about a comprehensive informational report of the IQWiG or multiple sources	31
**Research summaries**	About three-page summaries of systematic reviews or health technology assessment reports	71
**Supplementary elements**	Supplements to the central products, for example explanatory texts about organs or signs of illness, quizzes or pictorial material	5

The test readers assessed the extent to which the informational texts met their needs and demands, and how useful and understandable they were. The comprehensibility, the structure and the type of presentation were also part of the assessment [19]. Typically a package of four health information products composed by the IQWiG were assessed per focus group (Table 1) [9,25]. The IQWiG provided test versions of their informational products; the edited final versions are available on their website [16].

The test readers were recruited through bulletins, notices in the patient university newsletter and announcements during seminars and educational events. After voluntarily announcing their interest at the patient university’s office, the potential participants received an informational letter on the test procedure and conditions. Besides, they were asked to fill in a short questionnaire on characteristics such as age, gender, nationality, their education and profession, as well as health issues (membership of a self-help group, whether they suffered from a chronic illness or disability). Balanced groups of five participants were then chosen considering these characteristics and personal relevance on the health issues addressed by the informational materials (Table 2). A week prior to the focus groups, the texts were sent to the selected test readers. They were instructed to read the information carefully and note any open questions, unclear wordings or problems understanding the text. The test readers were told just after the whole discussion process that the IQWiG was the author of the evaluated texts. The focus groups took place in the rooms of the patient university and lasted between two and three hours. The participants reveived an allowance of €60.

**Table 2:** Characteristics of the test readers (N = 94)

**Characteristic**	**Women**	**Men**
Number	59	35
Age	47.0 years (Mean)	57.9 years (Mean)
	51 years (Median)	63 years (Median)
	15 – 82 years	15 – 79 years
Nationality		
German	96.6%	97.1%
Croatian	1.7%	2.9%
Slovakian	1.7%	-
**Educational level**		
No high school degree (yet)	10.2%	2.9%
General secondary school/polytechnic school	3.4%	2.9%
Intermediate secondary school	33.9%	8.6%
Vocational school degree	8.5%	5.7%
A-levels/general entrance qualification for university or university of applied science	13.6%	8.6%
Higher educational studies or training	30.5%	71.4%
**Professional group**		
Home-maker	3.4%	2.9%
Employee	50.8%	40.0%
(Blue-collar) worker	-	-
Self-employed	11.9%	8.6%
Civil servant	5.1%	37.1%
University student	3.4%	-
Pupil/trainee	23.7%	8.6%
Other	1.7%	2.9%
**Further attributes**		
Chronically ill	35.6%	45.7%
Member of a self-help group	5.1%	14.3%
Personal connection to the text discussed	31.9%	14.9%

An informed consent for participation in this study was obtained orally from all participants. The focus group discussion started with a brief introduction of the moderator and the participants, followed by information on the testing procedure and the recording conditions, which ensured confidentiality. Afterwards, the texts were intensively discussed on the basis of a structured discussion guide (Table 3). The focus groups were moderated by one of the authors (GS or MLD) who also took some notes and recorded the discussions with a digital recorder. Afterwards the audio data were transcribed by the moderators. A transcript of each discussed product was typically six to eight pages long, with anonymised original statements as well as paraphrased passages. The data were stored on the secured computer server of Hannover Medical School.

**Table 3:** Discussion guide with guiding questions for the discussion process

**Topics**	**Questions**
**What was your first impression, what did you notice?**	(Free-form description of first impressions without any guiding questions)
**Knowledge and understanding**	What is the central message of the text?
	What have you learnt?
	What do you find interesting about this information?
	How understandable is the text?
	How well are the facts explained?
	What aspects do you find important that are missing in the text?
	What concepts should be explained in the glossary?
**Language**	What is the writing style of this text?
**Use of numbers**	What effect do the numbers have in the text?
	Do they clarify the issues?
**Structure and readability**	Composition and structure
	Did the heading catch your interest and make you want to read on?
	Does the text answer what it says in the heading?
	Was the issue presented in an interesting way?
	Did it awaken your interest in reading on?
	What do you think about the length?
**Effects and anticipated effects**	Did reading this have any consequences for you?
	Will the text help to improve communication with doctors?
	Will the text help to improve communication with family and friends?
	Did the text increase your understanding of affected or ill people?
**Final assessment and recommendation**	Would you recommend the text to others?
	How would you rate the credibility of the author of this information?

### Material of the qualitative analysis

The data pool covered 107 discussion transcripts from 124 test readers in 27 focus groups. For the qualitative analysis some data had to be excluded: The discussion transcripts (n = 25) of the first six focus groups were categorised as pretest-material. Furthermore, the informational product type “supplementary elements” (Table 1) were not considered due to their high variability (regarding topic as well as type of text and material); therefore the transcripts (n = 3) of another focus group featuring only two other texts plus a supplementary element were excluded.

From the remaining 79 transcripts (discussed by 94 participants in 20 focus groups) a specifically targeted sample was drawn. We found that the first product dealt with in each session was the most intensely discussed. Therefore, the transcript of the first-discussed informational text from all 20 focus groups was analysed. In addition, another transcript from randomised groups was included until theoretical saturation was reached [26]. This process yielded 25 transcripts of 20 groups; the discussed informational products included 14 “fact sheets” and 11 “research summaries”. Fact sheets comprise three to six pages of easily understandable information in reference to a comprehensive report from the IQWiG or multiple sources, meant to give a quick overview of a topic. Research summaries are three-page summaries of systematic reviews or health technology assessment reports, meant as a kind of short answer to a scientific question, comparable to a news article [9]. The texts varied in their complexity and the difficulty of interpreting the given information. Most of the texts (88%) included numbers or proportions presenting e.g. study results or frequency of adverse effects or complications. Many texts (76%) indicated that in some aspects there were not sufficient reliable studies available or that the studies used were of partly ambiguous or uncertain evidence, and that therefore no clear recommendations could be given. The topics of the evaluated informational texts ranged from general topics (such as “using dietary supplements” or “expressing breast milk”) to specific interventions (such as “epidurals during childbirth” or “cognitive behavioural therapy” or “therapies for migraine”).

Ultimately user assessments of 25 informational products by 94 test readers were included in the analysis. The test readers are described in the results and in Table 2.

### Methods of the qualitative analysis

The qualitative analysis focused on elements of theoretical coding methods (open and axial coding) as well as content analysis techniques [26-29]. In the first step, a sample of six randomly selected transcripts was used to create a rough category scheme. Codings and paraphrases were formed very close to the texts. “Reaction patterns”, “formal textual criticism” and “dealing with research and evidence” crystallised as important thematic areas. In the next step, the codings were compiled into categories in a provisional scheme with initial differentiations. The scheme was extended through analysis of the other 19 transcripts. A second examination of all 25 coded transcripts resulted in the more detailed “axial” differentiation of the scheme into primary categories (PC) and first and second-order explanatory or descriptive subcategories (SC1 and SC2).

This category scheme was developed in a multi-step process whereby the procedure, the interim results and the category scheme were discussed with all but one of the participating authors (HB). The first transcript was coded independently by IH, DS, and MLD. All other transcripts were initially coded by IH. Unclear cases were solved by discussion with DS or MLD. Validity of the final results was checked by all authors. The analysing process was assisted by the text analysis software MAXQDA 2007©.

## Results

### Test readers of the study

The test readers were recruited from a pool of registered people who were interested in participation, primarily participants in the MHH patient university. Eight of the 94 test readers participated twice in the user testing. The gender distribution in the group was roughly three women to two men. The average age was 51 years (median: 57 years, range 15–82 years). 39.4% of the participants suffered from a chronic condition and 8.5% took part in a self-help group. More detailed descriptions including education, profession and nationality are shown in Table 2. In comparison with the German population, the test reader population had a higher proportion of women (51% vs. 62.8%) and fewer participants from other nations (8.8% vs. 3.2%). Besides, there were differences in the age structure (more elderly test readers over 45 and from 15–25 years old) and the educational level (more test readers with a higher level of education) between the test population and the wider German population.

### Category scheme for reaction patterns

**Figure 1:**
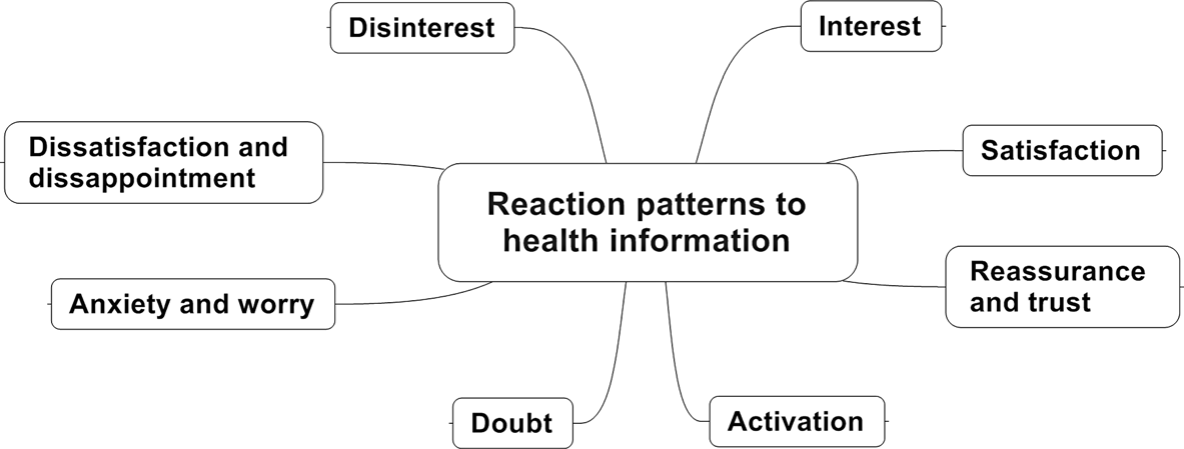
Category scheme for users’ reaction patterns to health information.

A category scheme for reaction patterns to health information was developed in close conjunction with the material in the written transcripts [24]. The scheme consists of eight primary categories (PC): the first four are more positively connoted, the second four more negatively: (i) interest, (ii) satisfaction, (iii) reassurance and trust, (iv) activation, and (v) disinterest, (vi) dissatisfaction and disappointment, (vii) anxiety and worry, and (viii) doubt (Figure 1). The explanations and descriptions for the reaction patterns were specified in 27 first-order subcategories (SC1) and 66 second-order subcategories (SC2).

Several of the test readers’ statements could be subsumed under two or more categories, and there was also some overlap between “positive” and “negative” reaction patterns, e.g. within the categories “interest” or “disinterest”. Some test readers said that a text would certainly be interesting for someone who was affected by its topic, but since they were not affected by it at the moment it did not matter to them. The “activation” of critical reflection, e.g. on the doctor’s attitudes and abilities, was also very close to a “doubting or mistrustful stance” towards the medical system. The statements were coded multiple times to capture these facets. Tables 4 and 5 describe the main categories and content of the reaction patterns and present selected quotations from the test readers. The subcategories cover the causes and details of the reaction patterns to health information, providing also an indication of readers’ expectations and informational needs (Additional file 1: Table S1 and Additional file 2: Table S2).

**Table 4:** Category scheme for test readers’ “positive” reaction patterns to health information with description and quotations

**Primary categories**	**Description**	**Quotations**
**Interest (i)**	Interest was expressed generally or tied to specific aspects such as being personally affected or having some connection to the topic like knowing an affected person. Other points of interest were particular contents or the structure of the text. This category has a clear connection to the discussion guide, with four questions about interest.	*It was interesting to learn what other men are doing. The presentation made me reflect on my own situation.* (T36, 37: NT08029).
**Satisfaction (ii)**	This reaction manifested itself in a positive overall impression of the informational item and in affirmative statements on the formal structure of the text, the comprehensibility of its explanations and the description of the data as a basis of evidence-based health information.	*I felt that they respected the reader. I felt that they took me seriously. I felt that it was written for me.* (T84: NT08053).
**Reassurance and trust (iii)**	This reaction pattern was prompted by a confirmation of the person’s own reading literacy as well as health literacy, for example. Information that supported the actions of people affected by the issue or that made them confident, e.g. by confirming their trust in medicine, were reassuring. Another factor was the feeling of security the readers got from acquiring knowledge or from a credible presentation of the information.	*If the antibiotic is taken responsibly, it’s a good thing. We don’t have to take it as often as we do.* (T84: NT08053)
		*I always thought, when older people fall, they always break a hip. This has taken away some of my fear.* (T137: NT08085).
		*Numbers are helpful and understandable. They’re reassuring too.* (NT08085).
**Activation (iv)**	This describes an activated stance or motivation on the part of the test readers at the level of thinking or action. It manifests itself in thoughts on the understanding of illness, for example, or in largely critical reflection, e.g. on the attitudes, abilities and responsibilities of the doctor and on the patient’s role (responsibility, co-determination) or on informational education (before informed consent), and the tested informational products and their research background. At the level of action we distinguished between activation beforehand (preparing the text), during (disagreements between the participants), and afterwards (resolutions, e.g. in dealing with the doctor, with their own family or with those affected with the illness). This category relates to questions in the discussion guide about the central message or about what the readers have learnt and what consequences it has.	*People should be more critical about taking food supplements.* (T33: NT08026).
		*It’s clear that the doctor and the patient are both responsible here.* (T84: NT08053).
		*Science hasn’t finished its research.* (T102: NT08065).

**Table 5:** Category scheme for test readers’ “negative” reaction patterns to health information with description and quotations

**Primary categories**	**Description**	**Quotations**
**Disinterest (v)**	This attitude became apparent in the lack of any personal relevance of the information (were not affected, had no personal connection or no consequences), the presentational style of the text or the context of the study. A loss of initial interest was also located in this category. Like interest, disinterest relates to the discussion guide, above all the aspect of whether the issue affects the readers personally.	*My first impression is also that the text is too long and imprecise for me, too diffuse* (T118: NT08101).
		*[…] As an ordinary reader I would have put it aside, but as an affected patient I would have read it* (T52, 49: NT08045).
**Dissatisfaction and disappointment (vi)**	Both the overall impression and individual aspects such as the composition and structure of the text, a lack of explanation, or the study content (e.g. lack of evidence, or an irritating presentation of the numbers or the results) led to dissatisfaction, expressed in critical remarks and expressions of displeasure. Remarks about a lack of medical background information and recommendations for action were also coded as disappointment and dissatisfaction.	*[I am uncertain] what the text is trying to tell me. With all my worries and needs and fears, I know exactly as much as I did before […] this text wouldn’t have helped me.* (T114: NT08097).
		*It’s too bad that this patient information wastes its chance of bringing people to a more informed level. I don’t see how reading this puts people in a position to make an actual judgment about the advantages and disadvantages of the procedure.* (T125: NT08077).
**Anxiety and worry (vii)**	Anxiety was caused by uncertainty about the readers’ own abilities, by the possibility that they might be affected by a serious illness, and by inconclusive data. The readers also had qualms about the therapeutic possibilities described which also prompted negative associations with the medical system. To some extent the readers distinguished their own anxiety from other readers’ possible worries about problematic effects and consequences. They discussed a distinction between feeling, e.g. the generation of fear, and potentially problematic action, e.g. discounting medication on their own authority.	*The statement that the preparation can have side effects doesn’t give the reader a lot of assurance* (T93: NT08069).
		*If the studies tell us so little, I wouldn’t feel safe starting such a therapy. I found everything so negative, I don’t know, am I the only one who felt that way?* (T66 NT08049)
**Doubt (viii)**	This sceptical reaction pertained to the study background (unclear procedures, low level of evidence, questionable conclusiveness, or conflicts of interest on the part of the researchers). Unclear or contradictory phrasing and explanations and an ambiguous presentation of results and numbers also led to mistrustful feelings. Here readers also emphasised the perspective of other readers, e.g. doubts about the comprehensibility for non-native speakers or older people. This category relates to the discussion guide among others in its questions on the use of numbers, the comprehensibility of the text and the assessment of the credibility of those responsible for the text.	*Weak effects, lots of open questions, […] what’s the point of this fact sheet if the studies aren’t far enough along or the whole research into Umckaloabo* (T96: NT08069).
		*I’m surprised at this phrasing: the result of studies so far – I wonder why, so far, they haven’t finished evaluating the studies?* (T133: NT08081).

Since the discussion guide placed great emphasis on questions of textual organization (e.g. composition and structure) and understandable language, the response to these formal aspects resulted in reaction patterns with a great many entries. However, there were agreeable and satisfied reactions to these features as well as displeasure and scepticism.

The test readers’ individual health-related knowledge and actions were addressed above all in response to the guiding questions about the central message of the text, what the readers learned, and the consequences of the information. Ultimately, the realization of one’s own uncertainty about what to do, for example, can lead to “anxiety and worry” but can also produce “reassurance and trust” when one’s own abilities and attitudes are supported or confirmed by the text. The readers often noted that their own behaviour could promote better handling of health problems. This confirmed some test readers in their previous actions. *I will continue to not buy it [author: nutritional supplements] and will try to convince my acquaintances to do so*. (T32, 34: NT08026)

Concerning their communication with doctors or other health professionals and their family or friend, test readers found the texts to be mostly helpful and supportive, despite some scepticism. But this required them to put themselves in the role of those affected and imagine the conversational situation, which was difficult for some test readers.

### Dealing with research and evidence

An important criterion for evidence-based information such as the tested IQWiG information is the relation to the state of scientific evidence. Regarding this aspect the test readers had different levels of knowledge about scientific studies. Their reactions differed greatly especially when the information offered a low level of evidence, no clear statement about options, or when they resulted in recommendations for further research. The reactions ranged from incomprehension, disillusionment and uncertainty to mistrust or even disbelief. *And then the study is explained in detail, but in the end it is said that it basically does not have a big impact*. (T115: NT08097) One reader was confused that *study results are shown in the text which are not convincing because of the low number of participants. Why are these results shown here?* (T50: NT08045). Also the scientific background of this information type was questioned: *Do studies always have to rely on evidence based results?* (T133: NT08081)

There was also doubt about the implementation of this knowledge in medical practices and about doctors’ ability to assess and use new knowledge. *Because it is said that investigations are ongoing, it is not yet clear, that there are these advantages and those disadvantages. As a patient one does not know at all whether my physician can really deal with that. […] The present evaluation does not have any value at all for me as a patient.* (T109: NT08093)

Many readers had difficulty to understand or interpret the numerical content of the cited studies in the informational products and the presented descriptions of risks or ratios. *Is 1 out of 50 children a little or a lot?* (NT08089) Some asked for more details or background, even pointing out misunderstandings in the ratios. Some could not deal with much numerical information. Nevertheless, the numerical data were largely seen as helpful. Readers were confused by a parallel presentation of numbers in absolute terms and percentages, and wanted the text authors to decide on one form.

Difficulties occurred especially when the information compared several divergent studies and their correspondingly complex results. A very detailed presentation was sometimes found to be a hindrance and the test readers suggested using graphs or tables for a better understanding.

Test readers mentioned also problems with ambiguous or imprecise statements like, “it may be possible”, “it could”, “it might be”. *The author does not specify*. [T132: NT080801).

The description of measures or procedures that were ultimately not recommended by the IQWiG due to unclear evidence or a lack of evidence confused some readers. They could not understand e.g. in a text on impetigo contagiosa why someone would describe measures only to conclude that they lacked any benefit. *If the use of antibiotic-free solutions and creams lack any benefit, why do they still write about it here?* (T131: NT09098) However, some other test readers found such statements helpful*. I could say that to my doctor, if he prescribes these solutions or creams*. (T130: NT09098). The texts prompted them to look more critically at possible therapies. But a reference to a lack of data could also evoke worries: *If the studies tell us so little, I wouldn’t feel safe starting such a therapy.* (T66: NT08049).

Many test readers had difficulties drawing conclusions for action from inconclusive data or unclear statements. In several cases they desired clear recommendations for action, or decision aids, particularly in texts on various therapy options. *As a patient I ask myself what can I actually do myself now?* (T132: NT08081).

Regarding the credibility of the information, some people expressed their doubt in two directions. They suspected that the authors of the informational product had a hidden agenda or that the researchers of the cited studies were partial or had conflicts of interest. The results themselves were also critically questioned concerning how reliable and how meaningful they actually were, and whether they could be applied to the German context. The many English references also caused some mistrustful responses.

## Discussion

### Reaction patterns as assessment framework

Our aim was to analyse readers’ reactions, assumptions and expectations, taking into account also their criticisms of the texts and suggestions for improvement. Though in the discussion guide for user testing there was no explicit question on expectations and informational needs, the test readers often mentioned these aspects. Therefore, the discussion transcripts were quite comprehensive on that. We extracted corresponding text passages with a focus on “formal textual criticism” and “dealing with research and evidence”, all summarized in a category scheme of reaction patterns.

#### ***Structure of the category scheme***

The scheme consists of eight primary categories of reaction pattern with further explanatory subcategories that vary by type and frequency (Tables 4, 5 and Additional file 1: Table S1 and Additional file 2: Table S2). These reaction patterns have “positive” (i–iv) and “negative” (v–viii) connotations. However, this should not be seen evaluative. For example, a negatively-connoted reaction pattern like doubt (viii) can express a “healthy scepticism” or a critical and reflective attitude towards information. This seems necessary for the assessment of a lot of information available on the internet. Nutbeam’s conception of health literacy and the achievement of a “critical health literacy level” explicitly aims at a critical assessment of health information [30]. “Dissatisfaction and disappointment” (vi), e.g. about the lack of definitive answers for action, reveals a clear sense of one’s own informational needs. In contrast “reassurance and trust” (iii) can be positively connoted but may also have more negative consequences if it leads to a lower level of engagement about illnesses.

The “negative” reactions can provide a clear orientation for changes or clarifications in the texts, e.g. in incomprehensible phrasings, suspicions of a hidden agenda or lack of clarity about the research background. Nonetheless, the “positive” reaction patterns should also be given critical consideration for the further development of such EBHI.

#### ***Factors influencing the test readers’ reactions***

The reaction patterns should be seen in the light of various influencing factors, including the test readers’ age, gender, and life situation, experiences with illness, attitudes, values and emotions. Even when they did not explicitly articulate their individual background, it influenced their assessment of the texts subliminally and contributed to the formation of their reaction patterns. The influence of journalism and advertising should also be taken into account. However, this was not a focus of this study.

#### ***Reaction patterns “interest” and “disinterest”***

Whether test readers had a personal connection with the informational issues was particularly relevant for “interest” (i) and “disinterest” (v). There was less interest in the information so long as the measure had no personal relevance for the reader. Hence, in many cases the reaction pattern was due to the test readers’ situation rather than the text. However, when the presentation of the information failed to appeal to readers, this could also contribute to a loss of initial interest.

#### ***Reaction patterns “satisfaction” and “dissatisfaction and disappointment”***

There were various aspects of the reaction pattern “satisfaction” (ii), including a positive overall impression and a good formal construction of the text. It also made a difference whether the readers felt respected. “Dissatisfaction and disappointment” (vi) in contrast occurred when the text provided insufficient support and when background information or recommendations were missing. At this point it is apparent that lay people are not familiar enough with the principles of EBHI. These principles include abstaining from recommendations so as not to steer the reader in a particular direction [6]. Satisfaction and dissatisfaction also depended on the individual test readers’ need for information. Here there were two distinct mutually exclusive tendencies: the frequently-expressed desire for more background information and more detail, and even numerical information, and the contradictory demand for concise presentation, which was also voiced in the focus groups.

#### ***Reaction patterns “reassurance and trust” and “anxiety and worry”***

Besides the message of the text, the reaction patterns “reassurance and trust” (iii) and “anxiety and worry” (vii) also showed a clear connection to the readers’ own abilities and their limitations. A confirmation of their own competence could convey assurance. However, it caused difficulties when readers came up against the limits of their own health literacy or reading literacy and when they were overwhelmed, for example with complicated and complex texts. This could motivate them to involve themselves more in the topic or to learn more, but it could also reinforce an inner insecurity.

The category “anxiety and worry” provides important indications of language or informational content that causes fear or anxiety. Non-alarmist and non-patronising language is a linguistic criterion of EBHI [12]. Describing the complications or adverse effects of diagnostic or therapeutic measures can be a tightrope walk between informing the readers truthfully about benefits, risks and problems and the danger of trivializing them. Hence, the worry was voiced in the focus groups that giving a false impression could lead to problematic consequences like patients discontinuing medication without consulting their doctor or not taking symptoms seriously.

#### ***Reaction patterns “activation” and “doubt”***

Even though the categories “activation” (iv) and “doubt” (viii) do not represent complementary concepts like the other reaction patterns, critical examination of the texts revealed a particular connection between doubt and activation to (critical) thought and reflection. The category “activation” is meant to capture that the test readers felt motivated to think or act by reading the texts. The connection with “doubt” is visible especially in activation to reflect on “the current state of research” or “health information and education”; critical thoughts arise also with respect to the presentation of studies and their research background and credibility. This is demonstrated also by the subcategories in Additional file 1: Table S1 and Additional file 2: Table S2.

In the context of activation to think or act, readers further mentioned aspects such as coping with one’s own illness, dealing with those affected, and the role of doctor and patient. The individual responsibility to act health-consciously or to actively participate in the healing process was also raised as an issue in this regard. This was seen as a duty to some extent, and also as an opportunity and a right to co-determination.

Readers also saw the texts as a challenge to become more active themselves. They felt that their impression that people can accomplish something themselves was confirmed. This contrasted with their impression of having to make any potential decision alone. Yet the dominant impression was that the information confirmed and supported them and that they gained competence in dealing with doctors and the health system. The readers felt better able to take on an active role in a doctor–patient partnership and to ask questions and express criticism. Their engagement with the state of research and evidence prompted them to ask questions about doctors’ ability to deal with uncertainties and gaps in the research, though in some readers it also seemed to reinforce their own insecurity as to whether they would be in good hands in case of anything serious.

The category “doubt” can also be interpreted as the expression of scepticism or mistrust. It was often voiced about an unclear presentation or about the basis of research.

### Dealing with research and evidence

Many test readers were not used to understanding EBHI. References to a low degree of evidence for certain data and reticence about making recommendations led to critical assessments of the texts; but it sometimes also led to greater trust in the quality of the information. Evidence-based decision-making requires an assessment of the potential harms and benefits on the basis of scientific studies [6,12]. Test readers mostly described the references to studies as helpful and interesting, but the exact presentation of numbers and results led to confusion and difficulties in understanding the text. The description of medical or care-giving measures that have not been proven to have any recognizable benefit but are used in practice was met with incomprehension. EBHI are also meant to help consumers to critically evaluate offered health measures and to notice possible misuse.

Moreover, as is well known, transferring and applying results from a collective study to one’s personal situation, and thus evaluating the risks for oneself, is a challenge – and this holds not just for lay-people but also for doctors and other health professionals [31].

Similar assessments of EBHI have been described by Glenton et al. They also report that many users find the personal descriptions of other affected patients to be helpful and interesting. They discuss whether and how such patient stories or narratives can be integrated into EBHI to illustrate research findings [15]. Even if so far there is insufficient experience with using such narratives in EBHI [10], these personal reports can make scientifically-based information more accessible to some readers [15]. There are some indications, including from IQWiG’s health information, that some readers give more credibility to the experiences of others who are personally affected or that come from their own personal environment than they do to scientific studies [20,32]. However, IQWiG texts containing narrative segments were not part of our sample.

As the user testing showed, many readers had difficulties distinguishing between the construction and statements of the text itself and the findings of the underlying studies. Especially under the categories “doubt” and “dissatisfaction and disappointment” readers spoke about methods, such as the criteria for the inclusion of studies, the applicability of the findings and the way these uncertainties are dealt with in practice.

Hence even without specific knowledge of the background of EBHI test readers touched upon the limitations and problems of EBM, sometimes questioning whether conclusions generally have to be based on evidence-based results. An increased awareness of the scientific background could probably help readers to deal with disappointment or anxiety over uncertain evidence. Readers were troubled by vague statements and were frustrated by gaps in current research or insufficient evidence. Yet even relatively well-established statements could raise questions if the findings did not match users’ personal experiences with their doctor’s treatment or messages from the media.

On the one hand, with written information the user has no conversational partner who can give direct feedback on things the reader may not have understood or on their anxiety or uncertainty. On the other hand, it may be easier to offer a thorough and balanced presentation of the data about and the background to the illness by writing rather than through direct contact with a doctor or counsellor. But even in a doctor–patient relationship the communication of uncertainties is a great challenge [33,34]. Anyway, it is important to investigate how any uncertainties influence the users’ behaviour and their search for additional information. Another research question is how the understanding of evidence-based knowledge of users and professionals can be improved. This is particularly important due to the growing amount of information available. In addition, we need more research on the role and value of health information in general [8,35,36] with a specific focus on the internet [37,38].

### Limitations and further research

In our study the users’ assessment is based on their reading of materials that often did not relate to their present situation – their assessment takes place under “artificial” conditions. This situation cannot be compared with the everyday conditions in which people have some specific reason to seek out information. Moreover, in cases of illness, patients’ ability to assess information and make decisions might be compromised. However, the users of online health information are also searching for information for others, so information-seeking individuals personally unaffected by the condition are also major users of health information.

The composition of test readers affects various user needs and the adaptation of health informational texts to these needs. IQWiG’s health information is intended for a broad audience. The participants in the user testing came from different social backgrounds, but do not exactly represent a cross-section of the German population. In further user testings we have increased efforts to recruit and include test readers with a low level of education, and from younger age groups in particular. Nevertheless, it is credible that the defined categories will cover the main range of reaction patterns; there may be just slight changes in their form and their weighting.

Additionally, further studies could concentrate on the following aspects: recruitment of test readers with a specific interest in a given topic (e.g. people who are affected by the illness described in the text); explicit questions on e.g. the users’ informational needs, their knowledge of scientific research and EBM, or their level of health literacy.

## Conclusions

This study on the assessment of EBHI from the users’ perspective illustrates the range of readers’ reactions to and perceptions of the texts. It also partly reveals what the readers’ expectations and informational needs are and which aspects could be considered in the production of this information type. The spectrum of reactions and differences in health and scientific literacy raise the question whether different types of EBHI for different target groups could better meet the informational need of the users: more research is needed here.

In any case, a central element of quality control for EBHI is the involvement of different user groups in its development and evaluation. That can help to uncover messages that the authors did not intend and to find more suitable phrasings. It can also uncover unintended effects that a detailed description of illnesses and therapies or potential complications might have on the reader. These aspects can be discovered by ascertaining readers’ perceptions. Our category scheme identifies the spectrum of the users’ reactions towards EBHI. It amplifies existing studies and evaluative models. Those studies have proposed criteria – besides presentation and applicability – such as emotional answers, gain in knowledge and competence [39] and communicative effectiveness [40]. Our focus on the users’ reaction patterns allows a closer look at how an unclear presentation or uncertain data are perceived. There is a need for further research that investigates how to acknowledge the following factors in the development of EBHI: 1) potential user reactions as outlined in our category scheme, and 2) how users understand EBHI and EBM.

### Practice implications

A clearer presentation of the basis of scientific research, e.g. on data acquisition and study quality, as well as the limits and possibilities of EBM and EBHI, might improve users’ understanding. EBHI should clarify the difference between insufficient evidence for the assessment of a measure and the demonstration that evidence to support a measure is lacking [9]. The presentation must also consider the uncertainties and “side effects” that the informational content might provoke among the readers. Our category scheme can help producers of EBHI to consider how users will potentially react and how they will deal with EBHI and the research background.

## Abbreviations

EBHI: Evidence-based health information; IQWiG: German Institute for Quality and Efficiency in Health Care (Institut für Qualität und Wirtschaftlichkeit im Gesundheitswesen); EBM: Evidence-based medicine.

## Competing interests

HB was the head of the Department of Health Information at IQWiG during the evaluations reported here and did not take part in those evaluations or analyses of primary data.

## Authors’ contributions

MLD and GS performed the data collection and interviews of the external user testing. IH performed the qualitative analyses, supported by MLD, GS and DS with whom she also discussed all results. Furthermore, IH wrote the draft version and revisions of the manuscript. MLD and DS helped to draft and rewrite the manuscript. HB participated in the design and coordination of the external user testing, and helped to revise the manuscript. All authors read and approved the final manuscript.

## Pre-publication history

The pre-publication history for this paper can be accessed here:

http://www.biomedcentral.com/1472-6963/13/405/prepub

## Supplementary Material

Additional file 1: Table S1 Category scheme on test readers’ “positive” reaction patterns to health information with subcategories. Click here for file

Additional file 2: Table S2 Category scheme on test readers’ “negative” reaction patterns to health information with subcategories. Click here for file
