# Supplementary material for: Evidence-based health information from the users’ perspective – a qualitative analysis
Source: BMC Health Serv Res. 2013 Oct 10;13:405. doi: 10.1186/1472-6963-13-405 (PMC3852570; doi:10.1186/1472-6963-13-405)
Supplement: Additional file 1: Table S1 — Category scheme on test readers’ “positive” reaction patterns to health information with subcategories. [file 1472-6963-13-405-S1.pdf]

Table S1: Category scheme on test readers’ “positive” reaction patterns to health information with subcategories

**Interest (i)**	**Reassurance and trust (iii)**
general interest	*Confirmation of their own abilities*
personal connection	– dealing with text and understanding it
content	– health-related knowledge and action
formal textual criteria	*Support and confidence*
	– trust in medicine
	– alleviates fear of illness
	– action for those affected
	Assurance
	– learning new things
	– credibility of the information
	– context and presentation of studies
**Satisfaction (ii)**	**Activation (iv)**
Overall impression	*concerning thought / reflection*
– positive	– understanding of illness
– tenor of the text	– therapy
– recommendation and utilisation	– personally concerned
formal textual criteria	– role as patient and user
– presentation and structure	– role of the doctor
– comprehensibility and explanations	– problems in everyday medical practice
context and presentation of studies	– current state of research
	– health information and education
	concerning behaviours / action
	– text preparation for the session and research
	– disagreements in the group discussion
	– health-related action
	– interaction in the doctor-patient relationship
	– dealing with one’s environment and those affected

The reaction patterns are placed on a grey background, the first-order subcategories are italicized and the second-order subcategories are preceded by dashes.
