# Supplementary material for: Evidence-based health information from the users’ perspective – a qualitative analysis
Source: BMC Health Serv Res. 2013 Oct 10;13:405. doi: 10.1186/1472-6963-13-405 (PMC3852570; doi:10.1186/1472-6963-13-405)
Supplement: Additional file 2: Table S2 — Category scheme on test readers’ “negative” reaction patterns to health information with subcategories. [file 1472-6963-13-405-S2.pdf]

Table S2: Category scheme on test readers’ “negative” reaction patterns to health information with subcategories

**Disinterest (v)**	**Anxiety and worry (vii)**
not appealing	*uncertainty*
no personal relevance	– limits of one’s own abilities
initial interest disappointed	– measures and illnesses described
context and presentation of studies	– trust in medicine (the medical system) impaired
	– lacking / insufficient data
	fears of critical effect
	– false impression, problematic consequences
	– fear and deterrence
**Dissatisfaction and disappointment (vi)**	**Doubt (viii)**
overall impression	*formal textual criteria*
– critical	– unclear contents and phrasings
– tenor of the text	– contradictions, doubtful or aspects
– no or limited recommendation	– doubts about comprehensibility
formal textual criteria	*context and presentation of studies*
– unnecessary or inappropriate detail	– general confusion
– mode of address and target group	– unclear methods
– presentation and structure	– unclear presentation of numbers and results
– indications of authors and sources	– questionable conclusiveness
– imprecise explanations	– lacking / insufficient data
– no clear statement or message	*credibility of the information*
missing / lack of pointers and background on	– hard to assess / trade-offs
– prevention	– hidden arguments and agenda
– research and studies	– lack of evidence and justification
– understanding of the illness	– doubts about the researchers’ independence
– diagnostics and therapy	
missing / lack of support	
– positive message	
– recommendations for action	
– interacting with those affected	
– decision aids	

- orientation in the health system

context and presentation of studies

- not convincing results or studies
- lack of evidence and classification
- presentation of results
- numbers are a hindrance or unhelpful
- way of presenting numbers

The reaction patterns are placed on a grey background, the first-order subcategories are italicized and the second-order subcategories are preceded by dashes.
